# Collagen-Based Interventions for Meniscal Tears: A Systematic Review of Clinical Outcomes, Safety, and MRI Findings

**DOI:** 10.7759/cureus.97929

**Published:** 2025-11-27

**Authors:** Mohamed Zahed, Mahmoud Elmesalmi, Ziad El Menawy, Nour Elnaggar, Ahmed Elkilany, Salam Elhanash, Mahmoud Odeh, Sara E Elbahnasawy, Sherif I Elhabbak, Mohamed Hesham Gamal

**Affiliations:** 1 Orthopaedics, John Radcliffe Hospital, Oxford University Hospitals National Health Service (NHS) Trust, Oxford, GBR; 2 Trauma and Orthopaedics, St George's University Hospitals, London, GBR; 3 Trauma and Orthopaedics, University Hospital of Wales, Cardiff, GBR; 4 Paediatric Burns and Plastic Surgery, Royal Manchester Children’s Hospital, Manchester, GBR; 5 Trauma and Orthopaedics, Manchester Royal Infirmary, Manchester, GBR; 6 Internal Medicine, Zayed Military Hospital, Abu Dhabi, ARE; 7 Microbiology, Faculty of Medicine, Zagazig University, Zagazig, EGY; 8 Orthopaedics and Trauma, Worthing Hospital, University Hospitals Sussex National Health Service (NHS) Foundation Trust, Worthing, GBR; 9 Trauma and Orthopaedics, St George’s University Hospitals National Health Service (NHS) Foundation Trust, London, GBR; 10 Trauma and Orthopaedics, Cardiff and Vale University Health Board, Cardiff, GBR; 11 Diagnostic Radiology, Menofia University Hospital, Menofia, EGY; 12 Surgery, Faculty of Medicine and Surgery, October 6 University, Giza, EGY; 13 Pharmacy, Banha University Hospitals, Banha, EGY; 14 Pharmacology and Therapeutics, Faculty of Pharmacy, Tanta University, Tanta, EGY

**Keywords:** arthroscopic matrix repair, collagen interventions, collagen supplementation, meniscal pathology, meniscal tears, meniscus implant, systematic review

## Abstract

Meniscal tears represent a significant clinical challenge worldwide, with traditional treatments ranging from conservative management to meniscectomy. Collagen-based interventions have emerged as promising alternatives, including collagen meniscus implant, arthroscopic matrix-based meniscus repair, and oral collagen supplementation. Despite their growing use, the comparative efficacy and safety profiles of these treatments are unclear. This systematic review aimed to evaluate and compare the clinical outcomes, safety, and structural changes of these three collagen-based interventions for meniscal pathology.

Following the Preferred Reporting Items for Meta-Analyses and Reviews (PRISMA) guidelines, we systematically searched five electronic databases for studies evaluating collagen-based interventions in adults with meniscopathy. The included studies reported functional outcomes, quality of life measures, or pain scores using validated instruments. Study designs included randomized controlled trials, case-control studies, cohort studies, and case series. Quality assessment was conducted using the Revised Cochrane Risk of Bias Tool, the Joanna Briggs Institute Critical Appraisal Checklist, and the Newcastle-Ottawa Scale.

A total of 27 studies involving 1,264 patients were included. The collagen meniscus implant demonstrated sustained functional improvement over extended follow-up periods with Lysholm scores ranging from 80 to 95 and subjective International Knee Documentation Committee (IKDC) scores of 77-95; however, MRI revealed predominant partial resorption (79%-92% of cases), frequent meniscal extrusion (68%-72%), and failure rates ranging from 1.5% to 40% (averaging 11%-12%). Arthroscopic matrix-based meniscus repair showed progressive improvement with subjective IKDC scores advancing from 77-79 at two years to 85-89 at 10 years, and Lysholm scores improving from 88-89 at two years to 92-93 at 10 years. MRI evaluations demonstrated favorable structural outcomes with 85% achieving Whole-Organ Magnetic Resonance Imaging Score (WORMS) scores ≤1, and lower failure rates (4%-21%). Oral supplementation provided rapid symptomatic relief within one to three months with significant improvements in pain (Visual Analog Score (VAS): 4.0) and quality of life (Knee Injury and Osteoarthritis Outcome Score (KOOS): 52.4). No major adverse events were reported. However, collagen meniscal implant (CMI) showed higher revision surgery rates.

Arthroscopic matrix-based meniscus repair (AMMR) appears most favorable for long-term outcomes in complex tears, demonstrating superior structural preservation and lower failure rates compared to CMI. CMI remains an option for partial deficiency despite high resorption rates, while oral supplementation provides a safe, conservative management option. However, these findings must be interpreted cautiously due to the absence of direct comparative studies and substantial heterogeneity. Robust head-to-head randomized controlled trials with standardized protocols are essential for evidence-based clinical decision-making.

## Introduction and background

The menisci are crescent-shaped fibrocartilaginous structures located in the knee joint [1]. They distribute load across the joint, absorb shock, provide stability, and maintain joint lubrication [2]. The meniscus consists primarily of water and extracellular matrix components, such as type I collagen, which is the predominant structural protein, glycosaminoglycans, and small amounts of type II collagen fibers [3,4]. The meniscus has three zones with different healing abilities based on blood supply, ranging from good healing in the outer zone to poor healing in the inner zone [4]. Given the regenerative capacity of meniscal tissue, injuries to these structures pose significant clinical challenges.

Among knee injuries, meniscal tears occur with high frequency across different age groups. In younger patients, tears typically result from acute trauma during sports or physical activities. However, older people typically develop tears from age-related changes in the meniscus [5]. The majority of meniscal tears occur in the avascular inner zone and thus do not heal. Patients typically experience a dramatic reduction in daily activities [6]. The management approach for meniscal tears depends on multiple factors. Meniscal injury treatments have improved dramatically over the years, encompassing both non-surgical methods and surgical options [7].

Treatment strategies can be broadly categorized into conservative management and surgical intervention, with the choice guided by tear characteristics and patient factors [6]. Conservative management is often used for patients with minor lesions. Non-surgical approaches mainly focus on symptom management and functional restoration. These options include physical therapy, pain medications, and activity modification. Recent advances in conservative treatment have explored oral collagen supplementation as an adjunctive treatment for meniscal pathology, due to its positive effects on healing and symptom reduction [8]. Type I, II, and III collagen peptides, often combined with glucosamine, chondroitin sulfate, and hyaluronic acid, may support meniscal healing through several mechanisms [9].

When conservative measures fail or are inappropriate, surgical intervention becomes necessary. Historically, meniscectomy was standard, but this accelerates degenerative changes and increases the risk of arthritis. [10]. Therefore, modern treatment prioritizes meniscal repair over meniscectomy to preserve long-term knee function [11,12].

Repairing the meniscus with a simple suture is a less aggressive surgical choice. Still, it has limited indications [13]. This paradigm shift has driven the development of meniscal replacement strategies that utilize both transplanted tissue and biological scaffolds for unreparable tears [14].

When repair is not feasible, meniscal replacement options include meniscal allograft transplantation (MAT) and meniscal scaffolds. MAT is indicated for patients who have undergone total or subtotal meniscectomy. In contrast, meniscal scaffolds are suitable only for partial meniscal loss, as they require the structural support of an intact rim and preserved anterior and posterior horns [15]. Regarding the scaffolds, there are three main options: the collagen meniscal implant (CMI), the Actifit (Orteq Sports Medicine, London, UK), which is a polyurethane scaffold, and the dimensionally printed scaffolds [16,17]. Comparative studies have shown that CMI and Actifit scaffolds achieve similar clinical outcomes in terms of pain reduction and functional improvement, with the choice of scaffold depending on the tear characteristics, patient factors, and surgeon preference [18].

Another emerging approach for meniscal replacement is arthroscopic matrix-based meniscus repair (AMMR). This technique combines meniscal suturing with collagen matrix wrapping and biologic augmentation using autologous bone marrow aspirate injection. This technique evolved from Henning's concept of creating a biological compartment with an optimal healing environment [19], initially using periosteum, but later replaced with collagen type-I matrix derived from porcine peritoneum. AMMR represents a meniscal wrapping approach that is fully arthroscopic and designed to enhance the healing of complex meniscal tears that would otherwise require meniscectomy [20]. Early clinical studies by Piontek et al. demonstrated relatively high survival rates and favorable functional outcomes at two and five-year follow-up [21].

Despite these treatment advances, a critical knowledge gap exists in the comparative understanding of collagen-based interventions for meniscal pathology. While CMI, AMMR, and oral collagen supplementation all utilize collagen as their therapeutic foundation, they differ fundamentally in mechanism of action, invasiveness, cost, and clinical indications. These approaches have been evaluated independently, but direct comparative evidence regarding their clinical efficacy, safety profiles, structural outcomes, and cost-effectiveness remains absent, leaving clinicians without evidence-based guidance for optimal intervention selection based on patient and tear characteristics.

Despite these treatment advances, questions remain regarding the comparative effectiveness in meniscopathy patients, particularly emerging interventions. In our study, we aim to conduct a systematic review of the efficacy and safety of collagen supplementation, AMMR, and CMI in preserving knee joint function, providing insights into the most effective interventions in various clinical scenarios.

## Review

Methods

This review has been conducted following the standards of the Preferred Reporting Items for Systematic Reviews and Meta-Analyses (PRISMA) guidelines [22].

The Review Question

What are the clinical outcomes, safety profiles, and MRI-documented structural changes associated with CMI, AMMR, and oral collagen supplementation in the treatment of meniscal tears in adults?

Literature Search

We searched five different electronic databases (PubMed, Web of Science, Scopus,cochrane, and Embase) using the search strategy of (Collagen OR Avicon OR Avitene OR Collastat OR Dermodress OR Pangen OR Zyderm OR Instat OR Helistat OR Collatamp OR Fibracol OR Puracol OR Promogran OR Biopad OR CollaCote OR CollaPlug OR CollaTape OR Zyplast OR Artecoll OR Cymetra OR BioGide OR Periocol OR CollaGuide OR OsseoGuard OR Verisol OR Fortigel OR Peptan) AND (Meniscopathy OR “Meniscal disease” OR “Meniscal pathology” OR “Meniscal lesion” OR “Meniscal disorder” OR “Meniscal degeneration” OR “Degenerative menisc*” OR “Meniscal injur*” OR “Meniscus tear*” OR “Meniscal tear*” OR “Meniscal Defect*” OR “Torn meniscus” OR “Meniscal derangement” OR “Meniscal syndrome” OR “Meniscus implant*” OR “Meniscal implant*”) with considering to answer the research question. A comprehensive search strategy for each database is presented in the supplementary material.

Inclusion Criteria

Adults (≥18 years) with meniscopathy (degenerative, traumatic, or symptomatic meniscal lesions). We included any collagen-based intervention, including collagen supplementation, collagen matrix, or collagen meniscus implant. We also included any studies reporting at least one of the following: clinical functional scores (IKDC, Lysholm, Tegner), quality of life (12-Item Short Form Health Survey (SF-12), SF-36), or pain (Visual Analog Score (VAS), Knee Injury and Osteoarthritis Outcome Score (KOOS)). Any study designs with the following were included: randomized controlled trials (RCTs), non-randomized controlled studies, cohort studies, case-control studies, and case series. We only included peer-reviewed articles published in English. No restrictions were assigned on the year of publication.

Exclusion Criteria

We excluded any non-English publications, conference abstracts without full-text availability, duplicate publications or overlapping datasets, case reports, and editorial studies or letters. Finally, any animal studies were excluded.

Critical Appraisal

Following the eligibility criteria and PRISMA guidelines, the included studies were independently assessed by both reviewers, with any discrepancies resolved through discussion until consensus was achieved.

Quality Assessment

We used three different quality assessment tools to assess the quality of the included study designs. First, we applied the Revised Cochrane Risk of Bias Tool for Randomized Trials (RoB 2) to the included RCTs [23]. RoB2 comprises five elements to be assessed: randomization process, deviations from intended interventions, missing outcome data, outcome measurement methods, and selective reporting of results. For case series studies, we used the Joanna Briggs Institute (JBI) Critical Appraisal Checklist [24]. The JBI Critical Appraisal Checklist for Case Series Studies evaluates methodological quality across 10 domains: clarity of inclusion criteria, standardization of condition measurement, validity of identification methods, consecutive participant inclusion, complete participant inclusion, reporting of demographics, reporting of clinical information, reporting of outcomes, site demographic reporting, and appropriateness of statistical analysis. Each domain receives a rating of "Yes," "No," or "Unclear," with studies included in the review if they demonstrate sufficient methodological quality based on overall evaluation. Finally, we employed the Newcastle-Ottawa Scale (NOS) for case-control studies [25]. The scale evaluates case-control studies across three categories: selection (adequate case definition, representativeness, control selection and definition), comparability (of cases and controls), and exposure (ascertainment methods, consistent methodology, and non-response rate). Studies receive up to nine stars in total, with quality classified as "Good," "Fair," or "Poor" based on the number of awarded stars.

Data Collection Process

Data extraction was performed systematically using standardized Excel (Microsoft, Redmond, WA) spreadsheets and RevMan 5.4 (The Cochrane Collaboration, London, UK) by two independent reviewers. The extracted data encompassed multiple domains, enabling comprehensive analysis. First, study-level characteristics were recorded, including study identification, geographic site, study design, inclusion criteria, primary outcomes or endpoints, and key conclusions. Second, baseline demographic and clinical data were captured for each study arm, including sample size, patient age, follow-up duration, gender distribution, operative side, underlying diagnosis, range of motion, and preoperative knee scores. Third, intervention-specific details were documented, including the type of collagen-based treatment (CMI, AMMR, or oral supplementation), any concomitant procedures (such as anterior cruciate ligament (ACL) reconstruction), surgical technique specifications, and supplementation protocols where applicable. Fourth, outcome measures were collected at multiple time points, including functional scores, quality-of-life assessments, pain measurements, structural imaging findings, and safety data, including failure rates and adverse events. All extracted data were cross-verified between reviewers, with discrepancies resolved through discussion and consensus.

Definition of Outcome Measures

Subjective International Knee Documentation Committee (IKDC) Score: A patient-reported outcome measure assessing symptoms, function, and sports activity in knee disorders. Scores range from 0 to 100, with higher scores indicating better knee function and lower levels of disability [26].

Lysholm Score: An eight-item questionnaire evaluating knee-specific symptoms, including instability, pain, swelling, stair climbing, and squatting. Scores range from 0 to 100, with scores above 90 considered excellent, 84-90 good, 65-83 fair, and below 65 poor [27].

Tegner Activity Scale: A numerical scale from 0 to 10 that rates activity levels based on work and sports participation. Level 0 represents sick leave or disability, while level 10 represents competitive sports at an elite level [28].

Visual Analogue Scale (VAS) for Pain: A continuous scale measuring pain intensity, typically ranging from 0 (no pain) to 10 (worst imaginable pain). Patients mark their pain level on a line, and the distance is measured to quantify the severity of their pain [29].

Knee Injury and Osteoarthritis Outcome Score (KOOS): A comprehensive knee-specific questionnaire evaluating five dimensions: pain, symptoms, activities of daily living, sport and recreation function, and knee-related quality of life. Each subscale is scored from 0 to 100, with higher scores indicating better outcomes [30].

Whole-Organ Magnetic Resonance Imaging Score (WORMS): A semi-quantitative MRI scoring system assessing 14 features across multiple knee compartments, including cartilage signal and morphology, bone marrow abnormality, meniscal integrity, ligaments, and synovitis. Higher scores indicate more severe structural pathology [31].

Genovese Criteria: An MRI classification system specifically designed to evaluate meniscal scaffold implants, assessing both morphology (size and shape) and signal intensity characteristics to grade implant integration and maturation [32].

Failure Rate: The proportion of patients requiring subsequent surgical intervention, experiencing significant symptom recurrence, or demonstrating treatment-related complications necessitating additional management during the follow-up period.

Results

**Figure 1:**
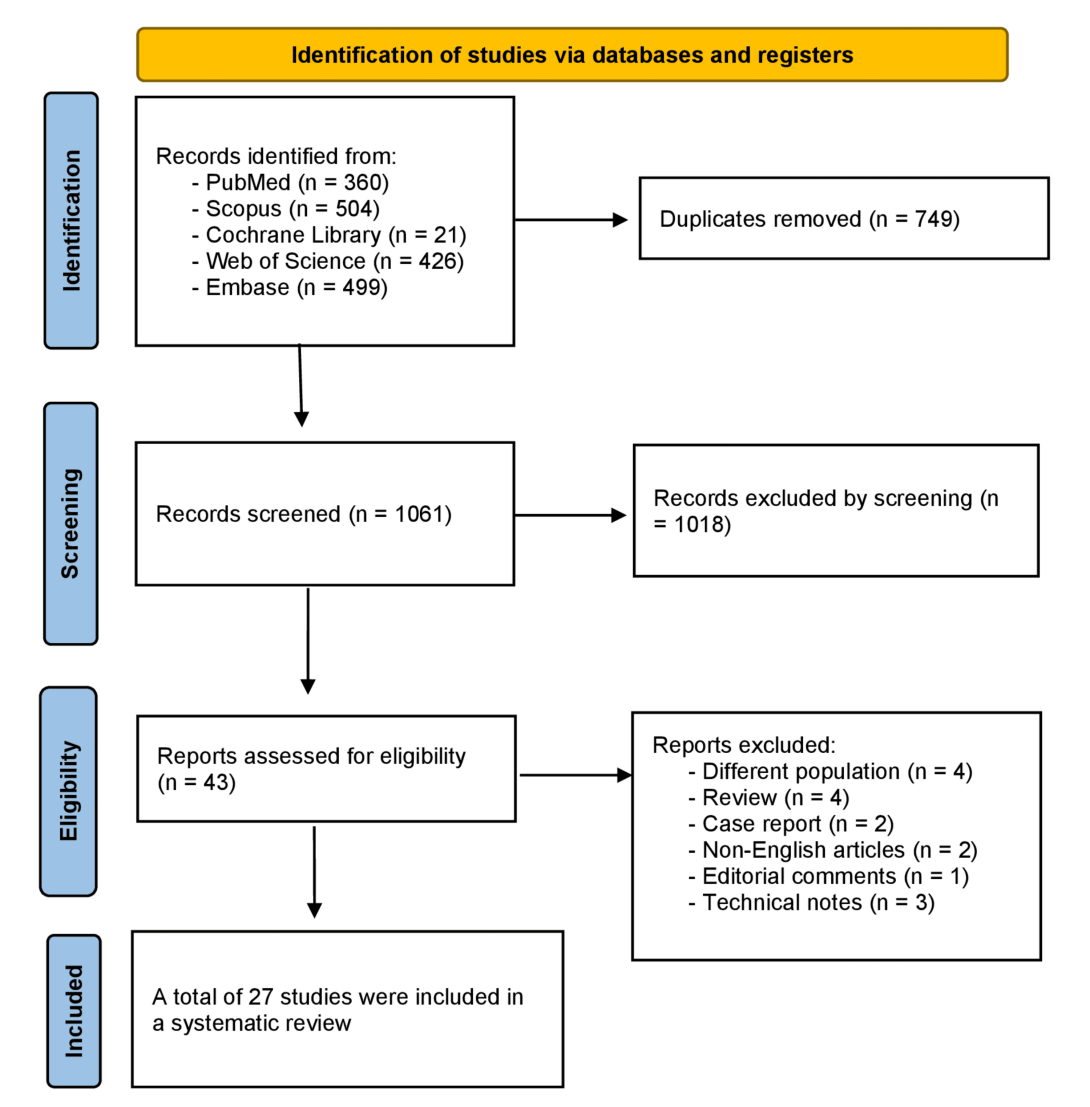
PRISMA flow diagram The included studies were References [9,10,18,20,32-54]. PRISMA: Preferred Reporting Items for Systematic Reviews and Meta-Analyses.

Using the search strategy across the five selected electronic libraries, the search yielded a total of 1,810 studies, of which 749 articles were excluded as duplicates. Of the remaining 1,061 studies, 1,018 were excluded during the initial title and abstract screening, 43 studies underwent full-text screening, and 27 studies met the eligibility criteria. They were included in this systematic review [9,10,18,20,32-54]. The flowchart of the strategy search for this systematic review has been summarized and presented in Figure 1.

Baseline and Summary of the Included Studies

The included studies enrolled patients from diverse geographic regions, with Spain, Switzerland, Turkey, Italy, the United States, the UK, Korea, and Poland being the most frequent contributors. The total number of patients reached 1264. Sample sizes varied substantially, ranging from small-scale randomized controlled trials (n=24-311) to retrospective and prospective case series enrolling between 21 and 156 patients. Across studies, the mean patient age typically ranged from the mid-30s to the mid-40s. Sex distribution was imbalanced in the majority of the studies, with male predominance noted in several groups. Body mass index (BMI) values consistently fell within the standard and overweight range. Both acute and chronic meniscal lesions were represented, with lesion morphology described as bucket-handle, horizontal, or complex tears in particular series. Lesion lengths were variably reported, although most averaged low, with a length of 30 mm.

At baseline, patients reported a considerable symptom burden, with quality-of-life scores ranging from the mid-30s to the mid-40s. Functional assessments (IKDC, Lysholm, Tegner) generally indicated reduced activity, while pain scores (VAS) averaged four to six, reflecting moderate pain. Overall, participants shared similar demographic and clinical features, showing moderate functional impairment and pain at study entry. The majority of studies employed prospective case series designs, ensuring comparability between the collagen-treated and control groups. The retrospective and prospective case series offered long observational windows, extending up to five to 10 years, thereby providing insight into the durability of collagen-based interventions. In contrast, the RCTs provided shorter follow-up durations compared to the observational studies.

The primary outcomes reported were broadly consistent across studies, centering on pain reduction, quality-of-life improvement, and functional recovery. Pain was assessed primarily via visual analogue scales (VAS), while quality of life and activity levels were captured through IKDC, Lysholm, and Tegner scoring systems. Longer-term series placed greater emphasis on sustained functional outcomes and complication rates, such as re-injury or progression to osteoarthritis. Interventions were mainly for symptomatic meniscal tears, from low-grade to complex or bucket-handle types, sometimes with ACL injuries. Across studies, collagen-based therapies have consistently shown improvements in pain, quality of life, and function, despite variations in sample size, design, and follow-up periods. Heterogeneity in study methods and outcomes highlights the need for standardized protocols and larger trials. Full details are shown in Tables 1, 2.

**Table 1:** Summary of the included studies References: [9,10,18,20,32–54] Abbreviations: NA, Not Available; ACL, Anterior Cruciate Ligament; CMI, Collagen Meniscus Implant; MRI, Magnetic Resonance Imaging; MR, Magnetic Resonance; USA, United States of America; UK, United Kingdom.

Study ID	Study Design	Recruitment Period	Country	Total number of patients	Follow-up Duration (years)	Primary Outcome	Collagen form of intervention	Meniscal Pathology Details/ Indication for surgery
Alfaro-Adrian et al. 2021 [50]	Randomized Controlled Trial	NA	Spain	24	0.25	Pain assessment and quality of life	Collagen supplementation - Sachet (Carticure Plus)	Meniscus injury
Genç et al. 2025 [9]	Randomized Controlled Trial	2024	Turkey	32	0.15	Pain assessment and quality of life	Collagen supplementation (type I, II , and III) - Sachets (Naturagen)	Meniscus tear (grade 1 and 2)
Bąkowski et al. 2023 [20]	Retrospective case series	April 2010 to November 2011	Poland	23	10	Clinical functional scores improvement	Collagen matrix wrapping+injection of bone marrow aspirate	Meniscus tear±ACL tears
Ciemniewska-gorzela et al. 2020 [53]	Retrospective-prospective case series	2010-2011	Poland	54	5	Clinical functional scores improvement	Collagen matrix wrapping + injection of bone marrow aspirate	Meniscus tear (Bucket-handle, Complex-horizontal, Complex-longitudinal, Radial, and Vertical tears)
Piontek et al. 2015 [21]	Prospective case series	April 2010 to November 2011	Poland	53	2	Improvement in knee function and meniscus related symptoms	Collagen matrix wrapping+injection of bone marrow aspirate	Combined and complex meniscal tears (horizontal and radial or longitudinal component)
Vicens et al. 2024 [44]	Retrospective case series	January 2017 to January 2023	Spain	21	3	Failure rate assessment	Collagen matrix wrapping	Chronic and complex meniscal tears (Bucket-handle, horizontal tears, and complex injuries)
Bulgheroni et al. 2010 [51]	Prospective case series	January 2001 to December 2003	Italy	34	5	Clinical functional scores improvement	Collagen Meniscus Implant	Medial meniscus tear OR persistent pain after meniscectomy (Meniscectomy, Bucket handle, Longitudinal, and Complex tears of the posterior horn of the meniscus)
Bulgheroni et al. 2014 [52]	Retrospective case-control	2001-2005	Italy	34	10	Clinical functional scores improvement	Collagen Meniscus Implant	Partial medial meniscus defects + ACL lesions
Bulgheroni et al. 2015 [18]	Prospective case-control	2001-2012	Italy	53	2	Clinical functional scores improvement	Collagen Meniscus Implant	Partial medial meniscal tear OR pain after partial medial meniscectomy (Chronic and acute lesions, Body and posterior horn, and Bucket handle)
Genovese et al. 2007 [32]	Prospective case series	March 2001 to June 2003	Italy	40	2	MRI assessment	Collagen Meniscus Implant	Medial meniscal lesions OR partial meniscectomy
Grassi et al. 2021 [54]	Prospective case series	April 2006 to September 2009	Italy	24	10	Surgery failure rate and Clinical functional scores improvement	Collagen Meniscus Implant	Partial lateral meniscal defects
Hirschmann et al. 2013 [33]	Prospective case series	2003-2011	Switzerland	67	1	Clinical functional scores and radiological improvement	Collagen Meniscus Implant	Previous subtotal medial or lateral meniscectomy
Kovacs et al. 2019 [34]	Retrospective case series	2005-2011	Switzerland	57	3-8	MR morphological characteristics and volume of CMI evaluation	Collagen Meniscus Implant	Meniscal defect
Linke et al. 2006 [35]	Randomized Controlled Trial	January 2001 to May 2004	Germany	60	2	Clinical functional scores improvement	Collagen Meniscus Implant	Subtotal degenerative or traumatic loss of the medial meniscus
Lucidi et al. 2022 [36]	Retrospective case-control	1998-2015	Italy	156	10	Long-term survivorship and risk factors for failure of CMIs	Collagen Meniscus Implant	Partial meniscal deficiency OR pain after partial meniscectomy
Monllau et al. 2011 [37]	Prospective case series	September 1997 to January 2000	Spain	25	10	Safety and efficacy of the device evaluation	Collagen Meniscus Implant	Large meniscus tear OR pain after partial medial meniscectomy (Complex, Horizontal, Bucket-handle , and Radial tears)
Reale et al. 2021 [39]	Retrospective case-control	2008 - 2011	Italy	47	10	Clinical functional scores improvement and failure of scaffold measurement	Collagen Meniscus Implant	Partial meniscal defect
Rodkey et al. 2008 [40]	Randomized Controlled Trial	NA	USA	311	5	Clinical functional scores improvement	Collagen Meniscus Implant	Medial meniscal injury OR previous partial loss of meniscus (Acute and Chronic)
Schenk et al. 2019 [41]	Prospective case series	NA	Switzerland	39	7-10	Clinical functional scores and radiological improvement	Collagen Meniscus Implant	Subtotal medial or lateral meniscectomy
Spencer et al. 2012 [42]	Prospective case-control	March 2008 to June 2010	UK	23	2	Pain relief	Collagen Meniscus Implant	Partial meniscectomy for resistant pain
Steadman and Rodkey 2005 [43]	Prospective case series	December 1995 to July 1996	USA	8	5-6	Clinical functional scores improvement	Collagen Meniscus Implant	Medial meniscus injury or previous partial medial meniscectomy (Acute or Chronic)
Whitehouse et al. 2016 [45]	Prospective case series	NA	UK	5	2	Clinical functional scores improvement	Autologous mesenchymal stem cells seeded onto a Collagen Meniscus Implant	Avascular medial meniscal tear (Bucket handle and Vertical flap tears)
Yoon et al. 2024 [46]	Randomized Controlled Trial	June 2021 to November 2022	Korea	36	1	Meniscus removing and defect-filling ratio	Collagen Meniscus Implant	Meniscal injury
Zaffagnini et al. 2006 [10]	Prospective case series	September 1997 to January 1999	Italy	8	6-8	Clinical functional scores improvement	Collagen Meniscus Implant	Meniscal tear or a previous meniscectomy involving the medial meniscus
Zaffagnini et al. 2011 [47]	Prospective case-control	October 1997 to March 2000	Italy	33	10	Clinical functional scores improvement	Collagen Meniscus Implant	Meniscal injury (Acute or Chronic)
Zaffagnini et al. 2012 [49]	Prospective case series	April 2006 to September 2009	Italy	24	2	Safety evaluation and clinical functional scores improvement	Collagen Meniscus Implant	Lateral meniscal tears or previous partial lateral meniscectomy
Zaffagnini et al. 2015 [48]	Prospective case series	April 2006 to September 2009	Italy, Spain, Germany, Switzerland, and Belgium	43	2	Safety evaluation and clinical functional scores improvement	Collagen Meniscus Implant	Partial lateral meniscal defects

**Table 2:** Baseline characteristics of the included studies References [9,10,18,20,32-54]. Abbreviations: ACLR, Anterior Cruciate Ligament Reconstruction; BMI, Body Mass Index; CMI, Collagen Meniscus Implant; ID, Identifier; IKDC, International Knee Documentation Committee; IQR, Interquartile Range; KOOS, Knee injury and Osteoarthritis Outcome Score; MSC, Mesenchymal Stem Cells; NA, Not Available; NO, Number; SD, Standard Deviation; VAS, Visual Analog Scale.

Study ID	Study Groups, (n)	Sex (male), NO.(%)	Age (years), Mean (SD)	BMI (kg/cm2), Mean (SD)	Meniscal lesion, NO.(%)		Lesion length (mm), Mean (SD)	Quality of life, Mean (SD)	Subjective IKDC, Mean (SD)	Lysholm score, Mean (SD)	Tegner activity scale, Median (IQR)	Pain score, Mean (SD)	
					Acute	Chronic							
Alfaro-Adrian et al. 2021 [50]	Collagen supplementation (n=6)	10 (83.3)	36.99 (4.9)	25.24 (3.02)	NA	NA	NA	36.46 (17.42)	NA	NA	NA	4.83 (1.47)	VAS
	Placebo (n=6)				NA	NA	NA	45.83 (14.61)	NA	NA	NA	3.83 (0.75)	VAS
Genç et al. 2025 [9]	Collagen supplementation (n=17)	6 (35)	41.7 (8.2)	28.3 (3.6)	NA	NA	NA	38.3 (6.9)	NA	NA	NA	5.3 (3.3)	VAS
	Placebo (n=15)	6 (40)	43.1 (13.7)	28.7 (4.2)	NA	NA	NA	37.9 (9.3)	NA	NA	NA	6.4 (2.3)	VAS
Bąkowski et al. 2023 [20]	Collagen matrix (n=16)	17 (74)	36 (11)	27 (3)	NA	NA	30 (6)	NA	44 (13)	66 (17)	NA	NA	
	Collagen matrix + ACLR (n=7)				NA	NA		NA	44 (15)	78 (13)	NA	NA	
Ciemniewska-gorzela et al. 2020 [53]	Collagen matrix (n=19)	16 (84.2)	42.2 (13.3)	25.2 (3.9)	NA	NA	29.7 (3.9)	NA	38 (13)	64.6 (36.04)	NA	NA	
	Collagen matrix + ACLR (n=20)	16 (80)	31 (11.2)	26.5 (2.6)	NA	NA	30.0 (5.8)	NA	45 (13)	67.33 (20.74)	NA	NA	
Piontek et al. 2015 [21]	Collagen matrix (n=48)	36 (75)	38.9 (13.5)	24.8 (3.5)	NA	NA	30.5 (3.7)	NA	44.5 (14)	66.1 (18)	NA	NA	
Vicens et al. 2024 [44]	Collagen matrix (n=21)	14 (66.7)	33.4 (10.25)	25.4 (3.25)	0 (0)	21 (100)	NA	NA	NA	NA	NA	NA	
Bulgheroni et al. 2010 [51]	Medial CMI (n=34)	25 (73.5)	39 (9)	NA	NA	NA	NA	NA	NA	58	2	NA	
Bulgheroni et al. 2014 [52]	Medial CMI (n=17)	13 (76)	32.9 (10.3)	NA	11 (64)	6 (36)	NA	NA	NA	57.3 (16.9)	3 (1–4)	5.35 (3.08)	VAS
	Medial meniscectomy (n=17)	13 (76)	34.3 (8.6)	NA	12 (71)	5 (29)	NA	NA	NA	61.1 (13.4)	3 (2–4)	3.84 (3.35)	VAS
Bulgheroni et al. 2015 [18]	Medial CMI (n=28)	19 (67.85)	38.7 (9.7)	NA	22 (78.57)	6 (21.43)	NA	NA	NA	58.4 (17.3)	2	NA	
	Actifit (n=25)	20 (80)	34.4 (11.4)	NA	7 (28)	18 (72)	NA	NA	NA	67.0 (15.7)	4	NA	
Genovese et al. 2007 [32]	Medial CMI (n=40)	27 (67.5)	41 (8.75)	NA	NA	NA	NA	NA	NA	NA	NA	NA	
Grassi et al. 2021 [54]	Lateral CMI (n=19)	16 (84)	37.1 (12.6)	23.7 (2.8)	4 (21)	15 (79)	45 (8)	0.55	NA	65	3	5.4	VAS
Hirschmann et al. 2013 [33]	Medial CMI (n=55)	47 (70.15)	35.5 (9.25)	NA	NA	NA	NA	NA	NA	69 (20)	3 (range 0–8)	4.4 (3.1)	VAS
	Lateral CMI (n=12)		36.55 (10.7)	NA	NA	NA	NA	NA	NA	63 (20)			
Kovacs et al. 2019 [34]	Medial CMI (n=48)	41 (71.9)	43.6 (11)	NA	NA	NA	NA	NA	NA	NA	NA	NA	
	Lateral CMI (n=9)			NA	NA	NA	NA	NA	NA	NA	NA	NA	
Linke et al. 2006 [35]	Correction osteotomy (n=16)	NA	41.6 (13)	NA	NA	NA	NA	NA	53	67	NA	5.2	Subjective pain
	Correction osteotomy + Medial CMI (n=23)	NA	41.8 (13)	NA	NA	NA	NA	NA	60.3	65.2	NA	4.9	Subjective pain
Lucidi et al. 2022 [36]	Medial/Lateral CMI (n=156)	117 (75)	42 (11.1)	24.9 (3.2)	34 (22)	122 (78)	NA	NA	NA	NA	NA	NA	
Monllau et al. 2011 [37]	Medial CMI (n=25)	20 (80)	31.23 (7.48)	28.1 (6.5)	20 (80)	5 (20)	NA	NA	NA	59.95 (15)	NA	5.5 (1.5)	VAS
Reale et al. 2021 [39]	Medial/Lateral CMI (n=25)	17 (68)	42.4 (10.4)	25.2 (3.9)	4 (16)	21 (84)	NA	NA	46.8 (16.7)	NA	3.2 (1.1)	4.4 (1.7)	VAS
	Medial/Lateral Actifit (n=22)	14 (63.6)	44.1 (12)	25.2 (3.4)	4 (18.2)	18 (81.8)	NA	NA	42.9 (15.9)	NA	2 (1.2)	5.4 (2.4)	VAS
Rodkey et al. 2008 [40]	Acute medial CMI (n=75)	65 (86.67)	40	NA	75 (100)	0 (0)	NA	NA	NA	64	NA	2.1	VAS
	Acute control (n=82)	67 (81.7)	40	NA	82 (100)	0 (0)	NA	NA	NA	59	NA	2.7	VAS
	Chronic medial CMI (n=85)	61 (71.76)	38	NA	0 (0)	85 (100)	NA	NA	NA	63	NA	3.7	VAS
	Chronic control (n=69)	50 (72.5)	39	NA	0 (0)	69 (100)	NA	NA	NA	56	NA	3.9	VAS
Schenk et al. 2019 [41]	Medial/Lateral CMI (n=39)	32 (82.05)	34.23 (9.8)	NA	NA	NA	NA	NA	NA	66 (20)	3.5 range (1–8)	4.3 (3.2)	VAS
Spencer et al. 2012 [42]	Medial/Lateral CMI (n=13)	19 (79.2)	32 (7.5)	NA	0 (0)	13 (100)	NA	31.5	48.1	61.8	3.7	60.3	KOOS
	Medial/Lateral Actifit (n=11)		39 (7.5)	NA	0 (0)	11 (100)	NA	27.8	42.1	56.5	3.8	56.7	KOOS
Steadman and Rodkey 2005 [43]	Medial CMI (n=8)	8 (100)	40 (10.3)	NA	1 (12.5)	7 (87.5)	NA	NA	NA	75.25 (17.3)	3.5 (3–4)	2.3 (1.14)	VAS
Whitehouse et al. 2016 [45]	MSC/Medial CMI (n=5)	4 (80)	35.5 (2.47)	25.3 (1)	5 (100)	0 (0)	30 (20.1)	NA	33.67 (31.17)	50 (30.17)		NA	
Yoon et al. 2024 [46]	Medial/Lateral CMI (n=19)	15 (78.95)	39.5 (14.3)	26.2 (2.88)	NA	NA	NA	34 (12)	45 (10)	NA	NA	5 (1.9)	VAS
	Medial/Lateral meniscectomy (n=14)	13 (92.85)	41.9 (12.4)	25.4 (2.88)	NA	NA	NA	36 (14)	46 (15)	NA	NA	4.2 (2.2)	VAS
Zaffagnini et al. 2006 [10]	Medial CMI (n=8)	8 (100)	33.25 (9.05)	NA	3 (37.5)	5 (62.5)	NA	NA	NA	NA	NA	5.1 (1.6)	VAS
Zaffagnini et al. 2011 [47]	Medial CMI (n=17)	17 (100)	40 (9)	25.24 (1.65)	7 (41.18)	10 (58.82)	36 (9)	33.5 (8.9)	NA	45.67 (8.08)	1 (0–2.5)	6 (1.6)	VAS
	Medial meniscectomy (n=16)	16 (100)	44 (8)	26.03 (1.88)	10 (62.5)	6 (37.5)	35 (8)	30 (3.25)	NA	42 (4.88)	1 (0.75–1.25)	7 (1.79)	VAS
Zaffagnini et al. 2012 [49]	Lateral CMI (n=24)	20 (83)	36.3 (11.5)	23.8 (2.6)	7 (29.17)	17 (70.83)	45.2 (8.1)	0.579 (0.28)	NA	64 (16.2)	3 (2-4)	5.52 (2.94)	VAS
Zaffagnini et al. 2015 [48]	Lateral CMI (n=43)	30 (70)	30.1 (12)	24.3 (3.4)	19 (44.2)	24 (55.8)	46 (11)	NA	NA	64.3 (18.4)	3 (2-4)	2.9 (2.5)	VAS

Quality Assessment

**Figure 2:**
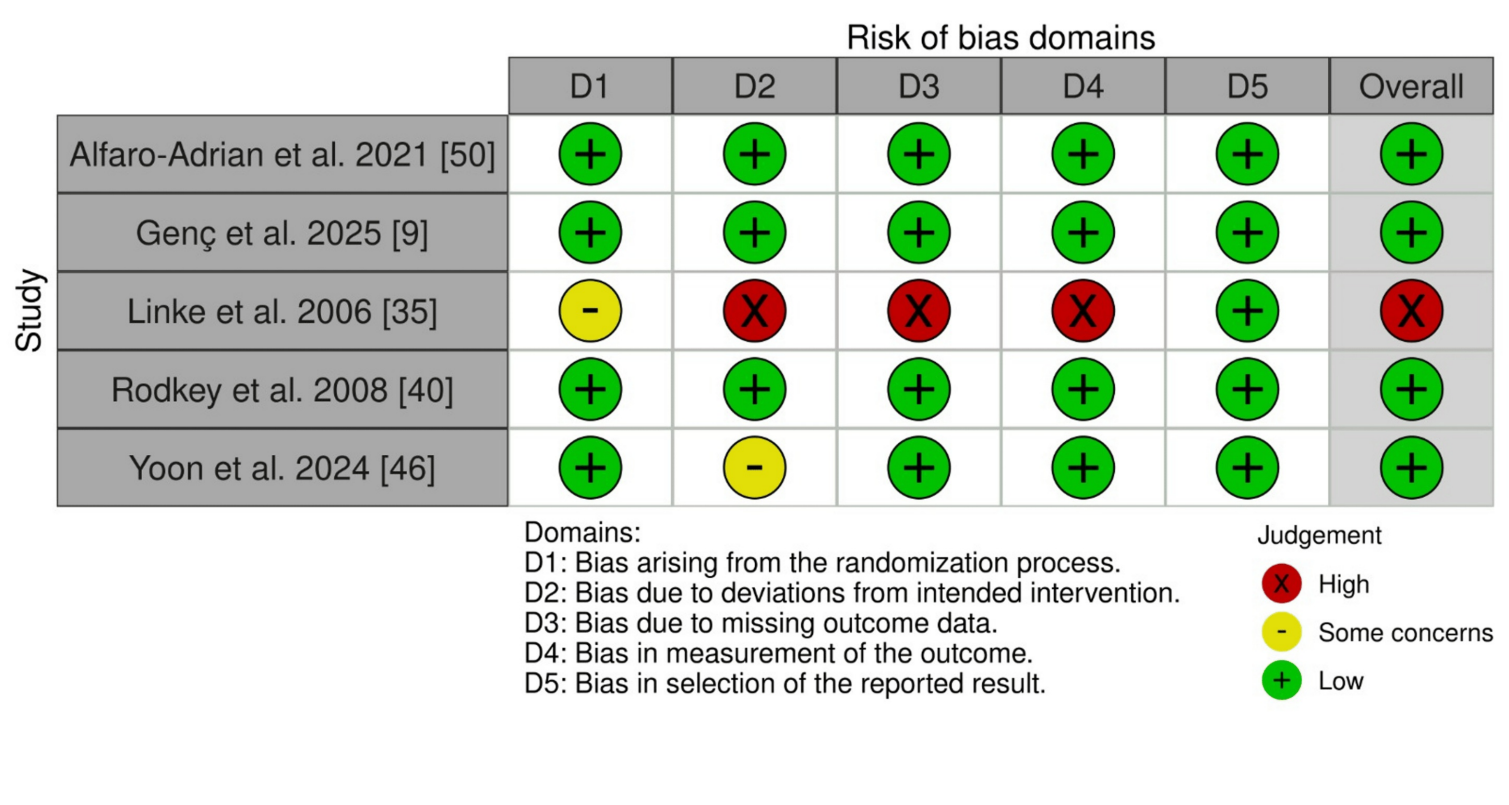
Risk of bias (ROB2) quality assessment of the randomized controlled trials (RCTs) References [9,35,40,46,50].

Five RCTs were assessed using the RoB 2 tool (Figure 2). Four studies (80%) demonstrated low risk of bias across all domains and received an overall "low risk" judgment. One study (Linke et al., 2006 [35]) was assessed as "High risk" overall, with specific concerns identified in deviations from intended interventions, missing outcome data, and issues with outcome measurement. Sixteen case series studies were included and assessed using the JBI Critical Appraisal Checklist (Table 3). Most studies clearly defined inclusion criteria. All studies measured the condition in a standard and reliable manner and employed valid identification methods. Common methodological limitations included incomplete inclusion of participants, with seven studies rated "No" for this domain, and inadequate reporting of site demographic information, with the majority of studies rated "Unclear." Additionally, statistical analysis was appropriate in all studies. Six case-control studies were evaluated using the NOS (Table 4). All studies were rated as "Good" quality overall. All studies received maximum stars for comparability of cases and controls. Four studies had adequate case definition, while only two demonstrated appropriate representativeness of cases. All studies adequately selected and defined controls. Regarding exposure assessment, all studies used appropriate ascertainment methods and applied consistent methodology for both cases and controls.

**Table 3:** Quality assessment of case series studies with the Joanna Briggs Institute (JBI) tool References: [10,20,21,32-34,37,41,43-45,48,49,51,53,54] D1   Were there clear criteria for inclusion in the case series? D2   Was the condition measured in a standard, reliable way for all participants included in the case series? D3   Were valid methods used for the identification of the condition for all participants included in the case series? D4   Did the case series have consecutive inclusion of participants? D5   Did the case series have complete inclusion of participants? D6   Was there clear reporting of the demographics of the participants in the study? D7   Was there clear reporting of clinical information of the participants? D8   Were the outcomes or follow-up results of cases clearly reported? D9   Was there clear reporting of the presenting sites’/clinics’ demographic information? D10  Was the statistical analysis appropriate?

Study ID	D1	D2	D3	D4	D5	D6	D7	D8	D9	D10	Overall decision
Bąkowski et al. 2023 [20]	Unclear	Yes	Yes	Yes	Unclear	Yes	Yes	Yes	Unclear	Yes	include
Bulgheroni et al. 2010 [51]	Yeses	Yes	Yes	Unclear	No	Yes	Yes	Yes	Unclear	Yes	include
Ciemniewska-gorzela et al. 2020 [53]	Yes	Yes	Yes	Yes	Unclear	Yes	Yes	Yes	Unclear	Yes	include
Genovese et al. 2007 [32]	Yes	Yes	Yes	Unclear	No	Yes	Yes	Yes	Unclear	Yes	include
Grassi et al. 2021 [54]	Yes	Yes	Yes	Yes	No	Yes	Yes	Yes	Unclear	Yes	include
Hirschmann et al. 2013 [33]	Yes	Yes	Yes	Unclear	No	Yes	Yes	Yes	Unclear	Yes	include
Kovacs et al. 2019 [34]	Yes	Yes	Yes	Yes	Unclear	Yes	Yes	Yes	Unclear	Yes	include
Monllau et al. 2011 [37]	Yes	Yes	Yes	Unclear	No	Yes	Yes	Yes	Unclear	Yes	include
Piontek et al. 2015 [21]	Yes	Yes	Yes	Yes	No	Yes	Yes	Yes	Unclear	Yes	include
Schenk et al. 2019 [41]	Yes	Yes	Yes	Unclear	Unclear	Yes	Yes	Yes	Unclear	Yes	include
Steadman and Rodkey 2005 [43]	Yes	Yes	Yes	Unclear	Unclear	Yes	Yes	Yes	Yes	Yes	include
Vicens et al. 2024 [44]	Yes	Yes	Yes	Yes	Yes	Yes	Yes	Yes	Unclear	Yes	include
Whitehouse et al. 2016 [45]	Yes	Yes	Yes	Unclear	No	Yes	Yes	Yes	Unclear	Yes	include
Zaffagnini et al. 2006 [10]	Yes	Yes	Yes	Unclear	Yes	Yes	Yes	Yes	Unclear	Yes	include
Zaffagnini et al. 2012 [49]	Yes	Yes	Yes	Yes	Yes	Yes	Yes	Yes	Unclear	Yes	include
Zaffagnini et al. 2015 [48]	Yes	Yes	Yes	Unclear	Unclear	Yes	Yes	Yes	Unclear	Yes	include

**Table 4:** Quality assessment of case control studies with the Newcastle-Ottawa Scale (NOS) tool References: [18,36,39,42,47,52] D1 Is the case definition adequate? D2 Representativeness of the cases D3 Selection of controls D4 Definition of controls comparability D5 Ascertainment of exposure D6 Same method of ascertainment for cases and controls D7 Non-response rate

Study ID	Selection				Comparability	Outcome			Overall
	D1	D2	D3	D4		D5	D6	D7	
Bulgheroni et al. 2014 [52]	*	*	*	*	**	*	*	*	Good
Bulgheroni et al. 2015 [18]	*		*	*	**	*	*		Good
Lucidi et al. 2022 [36]	*	*	*	*	**	*	*	*	Good
Reale et al. 2021 [39]	*		*	*	**	*	*		Good
Spencer et al. 2012 [42]	*		*	*	**	*	*		Good
Zaffagnini et al. 2011 [47]	*		*	*	**	*	*		Good

Clinical Efficacy

*Collagen meniscus implant*: Studies on collagen matrix implantation demonstrate sustained functional improvement across multiple validated assessment scales, with long-term follow-up data. In the Bulgheroni 2014 study, patients treated with medial CMI showed subjective IKDC scores of 85.7±14.4 at 10 years, representing sustained knee function over extended follow-up periods [52]. Lysholm scores demonstrated consistent performance with CMI achieving 94.1±8.2 at 10 years compared to 95.5±7.6 in the meniscectomy group [52]. The Bulgheroni 2010 study demonstrated Lysholm scores of 95 at 2 years and 92 at five years, with Tegner activity scores of 4.76 at two years and 5.32 at five years. The Bulgheroni 2015 study comparing medial CMI versus Actifit showed Lysholm scores for CMI of 92.5±8.5 at one year and 94.5±6 at two years, with Tegner scores of four at one year and five at two years [52]. Grassi et al. (2021) showed that lateral CMI achieves progressive functional improvement with Lysholm scores of 93 at two years, declining to 82 at 10 years [54]. Tegner activity scores decreased from 5 at two years to 3 at 10 years, with only 16% reported being pain-free [32]. Schenk et al. (2019) corroborated these findings with CMI treatment, achieving Lysholm scores of 95±6.5 at one year and 91±8 at seven years, while maintaining Tegner scores of 6 (4-9) at one year and 6 (3-10) at seven years [41]. Additional evidence from Steadman and Rodkey (2005) demonstrated progressive improvement in Lysholm scores from 89.38±9.5 at one year to 91.5±6.23 at two years, before stabilizing at 88.12±8.66 at five years [43]. KOOS analysis from Spencer et al.'s 2012 study revealed improvements in function scores from 49 at one year to 57 at two years, and quality of life scores advancing from 65 at one year to 71.8 at two years [42]. The Spencer et al. study showed Lysholm scores for CMI of 80 at one year and 82.9 at two years, with Tegner scores of 4 at one year and 5.2 at two years. Pain scores were 82 at one year and 88.8 at two years [42]. The Linke et al. (2006) study comparing correction osteotomy alone versus correction osteotomy+medial CMI showed that osteotomy alone achieved subjective IKDC scores of 84 at one year and 77 at two years, Lysholm scores of 94 at one year and 91 at two years, and pain scores of 1.1 at one year and 1.5 at two years [35]. The Zaffagnini (2006) study reported pain scores of 1.3±0.5 at one year, 1.1 ± 0.4 at two years, and 1.8±0.9 at seven years [10]. The Zaffagnini 2015 study for lateral CMI demonstrated Lysholm scores of 89.6±9.8 at one year and 93.2±7.2 at two years, Tegner scores of 5 (4-6) at one year and 5 (4-7) at two years, pain scores of 0.7±1.3 at one year and 0.3 ± 0.5 at two years [48].

*Arthroscopic matrix-based meniscus repair*: The collagen matrix studies demonstrate superior long-term clinical efficacy, with progressive functional improvement over extended follow-up periods compared to placebo. In the Bąkowski et al. (2023) study, patients treated with collagen matrix alone showed improvement in subjective IKDC scores from 79.3±14.63 at two years to 89±8 at 10 years, representing a clinically meaningful enhancement in knee function over time [20]. The Bąkowski et al. study demonstrated further improvement, with subjective IKDC scores progressing from 85±15.44 at five years to 89±8 at 10 years [20]. Similarly, Lysholm scores improved from 89.3±14.6 at two years to 93±9.75 at five years before stabilizing at 92±6 at 10 years. Finally, the combined collagen matrix with ACLR approach in the Bąkowski et al. study achieved subjective IKDC scores of 95±15 at 10 years [20]. The Ciemniewska-Gorzela et al. (2020) study corroborated these findings, with collagen matrix treatment achieving subjective IKDC scores of 77±8 at two years and 85±12 at five years, while Lysholm scores remained consistently high at 88±7 and 88.5±9.5, respectively [53].

*Collagen supplementation*: The Genç et al. (2025) study reveals a significant difference in quality of life scores, as assessed by the KOOS scale, between the collagen (52.4±7.8) and placebo (37.3±7.7) groups at two months, indicating superior improvement in quality of life for the supplementation group [9]. Both studies that employed the use of supplementation showed an improvement regarding pain reduction, showing better progress in the collagen group (4.0±2.8) compared to the placebo (5.6±2.1) [9,50]. The Alfaro-Adrián et al. (2021) study demonstrated improvements in quality of life in the collagen group, from 50±28.78 at one month to 79.17±20.79 at two months, before stabilizing at 77.5±28.84 at three months. Pain scores showed a reduction in the collagen group from 2±1.1 at one month to 1.5±2.35 at two months and 1.6±2.07 at three months [50]. It is noteworthy that the follow-up in both of the studies is short compared to the other included studies, which prevents the assessment of long-term outcomes.

*MRI and structural outcomes:* Multiple studies evaluated CMI using MRI with varying scoring systems. The Bulgheroni et al. (2010) study showed no further chondral degeneration according to the Yulish score at two and five years, with most patients maintaining normal or grade 1 cartilage. Subchondral bone edema was present in the femoral condyle in 10 patients, with a mean of 6.3 mm at two years and 7.1 mm at five years [51]. Grassi et al. (2021) reported that patients requiring joint replacement had the highest Yulish scores (grades 3-4) preoperatively and showed partial or total CMI resorption at two-year follow-up [54]. Using Genovese criteria, multiple studies documented implant changes over time [32]. Schenk et al. (2019) found complete resorption in 21% and partial resorption in 79% of patients, with 74% showing slightly hyperintense signal, 53% demonstrating bone marrow edema, and 68% exhibiting meniscal extrusion >3 mm [41]. Zaffagnini et al. (2012) reported that 87.5% of implants showed size reduction, with complete resorption in 12.5%. Meanwhile, 50% had a slightly hyperintense signal, and 37.5% achieved full maturation by the final follow-up [47]. Monllau et al. (2011) showed signal intensity progression from predominantly type 2 (64%) at final follow-up, with all cases demonstrating reduced volume and loss of distinguishable interface between implant and native tissue [37]. Genovese et al. (2007) demonstrated normal shape/size (type 3) in 83% of cases at 12 months, with signal intensity shifting from type 1 (35%) to type 2 (65%) over time [32]. Yoon et al. (2024) found no significant differences in total Whole-Organ Magnetic Resonance Imaging Score (WORMS) scores or cartilage variables between scaffold and control groups. Genovese grade showed a significant improvement in signal intensity (p=0.001) but not in morphology (p=0.063) [46]. Spencer et al. (2012) observed no chondral wear progression, with varying implant structural integrity from good in-fill to marked erosion, and persistent edema-like signal [42]. Kovacs et al. (2019) reported a hyperintense, inhomogeneous CMI signal in all patients with 93% showing meniscal extrusion, 100% having chondral defects, and 28% demonstrating bone marrow edema pattern on the femoral side [34]. Hirschmann et al. (2013) found 92% partial resorption, 90% slightly hyperintense signal, and 72% showing extrusion >3 mm [33]. The Bąkowski 2023 study demonstrated a statistically significant decrease in WORMS at 10 years compared to five-year follow-up across all patients. However, when analyzing AMMR and AMMR+ACLR subgroups separately, these differences were not significant. The difference between groups was only significant at the five-year follow-up [20]. The Ciemniewska-Gorzela et al. (2020) study showed a statistically significant increase in WORMS between two and five years. Subgroup analysis revealed that WORMS increased significantly between 24 and 60 months only in the AMMR+ACL group. The difference between groups increased and became significant at 60 months. Only one case scored >40 and was classified as early osteoarthritis [53]. The Piontek et al. (2015) study revealed 85% good meniscus outcomes based on WORMS classification (cumulative score ≤1) at two years. MRI showed non-homogeneous signal without meniscal tear (WORMS grade 1) in 76% of operated menisci, fully regenerating meniscal cartilage with homogenous signal (WORMS grade 0) in 11%, and WORMS grade 2 in 13%. The WORMS results did not correlate with clinical (IKDC 2000, Barrett) or subjective scores (IKDC subjective, Lysholm) [21]. Finally, the Vicens et al. (2024) study reported MRI findings of meniscal re-tear in one patient and complete ACL re-rupture in another case during follow-up [44]. Supplementation: Neither of the included studies performed an assessment using MRI [9,50].

Complications and Safety

The safety profile of CMI demonstrates acceptable complication rates with manageable adverse events across the reported studies. The Lucidi et al. (2022) extensive cohort study reported a failure rate of 12.2% in CMI procedures. The Lucidi study specifically documented 19 failures out of 156 procedures [36]. The Grassi et al. (2021) investigation documented a 26% failure rate in lateral CMI procedures over a 10-year follow-up [54]. Smaller series showed variable safety profiles, with Rodkey et al. (2008) reporting failure rates of 6.67% in acute medial CMI cases and 11% in chronic cases [40]. The Reale et al. (2021) study demonstrated a 20% failure rate at 10 years for CMI procedures. The Reale study, which compared CMI versus Actifit, showed failure rates of 20% for CMI and 22.7% for Actifit [39]. Long-term follow-up data from Zaffagnini et al. (2011) showed an 11.76% failure rate over 10 years [10], while Monllau et al. (2011) reported an 8% failure rate [37]. Whitehouse et al. (2016) documented higher failure rates of 40%, though this was attributed to the small sample size and experimental nature of the combined approach [45]. Additional safety data from various CMI studies showed: Hirschmann et al. (2013) with a 1.5% failure rate [33], Spencer et al. (2012) with an 11.1% failure rate [42], Zaffagnini et al. (2012), with a 4.17% failure rate [47], and Zaffagnini et al. (2015) with an 11.6% failure rate [48]. The Rodkey et al. (2008) study demonstrated failure rates of 6.67% for acute CMI and 6.1% for acute controls [40], while chronic CMI had 11% failure rate compared to 26% for chronic controls [40]. The Zaffagnini et al. (2011) study showed failure rates of 11.76% for CMI and 12.5% for meniscectomy [47]. No major complications such as infection, severe inflammatory reactions, or material-related adverse events were systematically reported across the included studies. The sustained long-term follow-up periods spanning up to 10 years indicate that patients remained available for assessment without experiencing severe adverse events that would preclude continued participation [47]. The safety profile of collagen matrix treatments demonstrates acceptable risk levels with manageable complication rates across the included studies. The Ciemniewska-Gorzela et al. (2020) study reported a failure rate of 21.1% in the collagen matrix group [53]. Specific failure rates for AMMR studies included: Ciemniewska-Gorzela et al. (2020), with a 12% failure rate for collagen matrix alone [53], and Piontek et al. (2015), with a 4.17% failure rate [38], and Vicens et al. (2024) with a 9.5% failure rate [44]. The long follow-up periods reported in these studies, up to 10 years, indicate that patients remained available for assessment and did not experience severe adverse events [20,51]. However, matrix treatments require surgical intervention with inherent risks including arthroscopic complications, anesthesia, and infection. Despite these considerations, the sustained long-term improvements suggest a favorable risk-benefit profile for appropriately selected patients [38]. The study results reported adverse events, side effects, or safety concerns during the three-month supplementation period. All participants completed the survey without any dropouts. To achieve maximum safety, all patients with allergies to the excipients or the product were excluded, resulting in no complications or side effects [9,50]. Despite these results, which confirm safety, they should be interpreted cautiously due to the small sample sizes and short follow-ups. A summary of all the previously extracted outcomes is presented in Table 5.

**Table 5:** Summary of the extracted outcomes References: [9,10,18,20,32–54] NA, Not Available; ACL, Anterior Cruciate Ligament; ACLR, Anterior Cruciate Ligament Reconstruction; CMI, Collagen Meniscus Implant; MRI, Magnetic Resonance Imaging; MR, Magnetic Resonance; USA, United States of America; UK, United Kingdom; BMI, Body Mass Index; IKDC, International Knee Documentation Committee; IQR, Interquartile Range; VAS, Visual Analogue Scale; KOOS, Knee Injury and Osteoarthritis Outcome Score; MSC, Mesenchymal Stem Cells; SD, Standard Deviation; NO, Number.

Study ID	Study Groups, (n)	Quality of life, Mean (SD)	Subjective IKDC, Mean (SD)	Lysholm score, Mean (SD)	Tegner activity scale, Median (IQR)	Pain score, Mean (SD)	Failure rate, NO.(%)
Alfaro-Adrian et al. 2021 [50]	Collagen supplementation (n=6)	1 month: 50 (28.78) 2 months: 79.17 (20.79) 3 months: 77.5 (28.84)	NA	NA	NA	1 month: 2 (1.1) 2 months: 1.5 (2.35) 3 months: 1.6 (2.07)	NA
	Placebo (n=6)	1 month: 55.21 (22.51) 2 months: 77.08 (14.61) 3 months: 72.5 (23.63)	NA	NA	NA	1 month: 2.17 (0.98) 2 months: 0.83 (0.41) 3 months: 0.83 (0.45)	NA
Genç et al. 2025 [9]	Collagen supplementation (n=17)	2 months: 52.4 (7.8)	NA	NA	NA	2 months: 4 (2.8)	NA
	Placebo (n=15)	2 months: 37.3 (7.7)	NA	NA	NA	2 months: 5.6 (2.1)	NA
Bąkowski et al. 2023 [20]	Collagen matrix (n=16)	NA	2 years: 79.3 (14.63) 5 years: 85 (15.44) 10 years: 89 (8)	2 years: 89.3 (14.6) 5 years: 93 (9.75) 10 years: 92 (6)	NA	NA	NA
	Collagen matrix+ACLR (n=7)	NA	2 years: 82.67 (24.8) 5 years: 85.67 (14.7) 10 years: 95 (15)	2 years: 90 (9.19) 5 years: 83.67 (20.2) 10 years: 90 (11)	NA	NA	NA
Ciemniewska-gorzela et al. 2020 [53]	Collagen matrix (n=19)	NA	2 years: 77 (8) 5 years: 85 (12)	2 years: 88 (7) 5 years: 88.5 (9.5)	NA	NA	4 (12)
	Collagen matrix + ACLR (n=20)	NA	2 years: 80 (10) 5 years: 86 (14)	2 years: 88 (11) 5 years: 90 (10.5)	NA	NA	
Piontek et al. 2015 [21]	Collagen matrix (n=48)	NA	2 years: 85.77 (15.19)	2 years: 78.6 (16.4)	NA	NA	2 (4.17)
Vicens et al. 2024 [44]	Collagen matrix (n=21)	NA	3 years: 73.3 (8.08)	NA	3 years: 1.9 (0.97)	NA	2 (9.5)
Bulgheroni et al. 2010 [51]	Medial CMI (n=34)	NA	NA	2 years: 95 5 years: 92	2 years: 4.76 5 years: 5.32	NA	NA
Bulgheroni et al. 2014 [52]	Medial CMI (n=17)	10 years: 0.9282 (0.1285)	10 years: 85.7 (14.4)	10 years: 94.1 (8.2)	10 years: 6 (5–6)	10 years: 1.47 (1.87)	NA
	Medial meniscectomy (n=17)	10 years: 0.9499 (0.1116)	10 years: 88.1 (7.2)	10 years: 95.5 (7.6)	10 years: 6 (5–6)	10 years: 1.35 (1.62)	NA
Bulgheroni et al. 2015 [18]	Medial CMI (n=28)	NA	NA	1 year: 92.5 (8.5) 2 years: 94.5 (6)	1 year: 4 2 years: 5	NA	NA
	Actifit (n=25)	NA	NA	1 year: 87.4 (13) 2 years: 90.3 (13.1)	1 year: 5 2 years: 5	NA	NA
Genovese et al. 2007 [32]	Medial CMI (n=40)	NA	NA	NA	NA	NA	NA
Grassi et al. 2021 [54]	Lateral CMI (n=19)	2 years: 0.91 10 years: 0.74	NA	2 years: 93 10 years: 82	2 years: 5 10 years: 3	2 years: 1.5 10 years: 3.1	5 (26)
Hirschmann et al. 2013 [33]	Medial CMI (n=55)	NA	NA	1 year: 93 (9)	1 year: 6 (2–10)	1 year: 1.9 (2.2)	1 (1.5)
	Lateral CMI (n=12)	NA	NA	1 year: 90 (9)		1 year: 2.2 (2.2)	
Kovacs et al. 2019 [34]	Medial CMI (n=48)	NA	NA	NA	NA	NA	NA
	Lateral CMI (n=9)	NA	NA	NA	NA	NA	NA
Linke et al. 2006 [35]	Correction osteotomy (n=16)	NA	1 year: 84 2 years: 77	1 year: 94 2 years: 91	NA	1 year: 1.1 2 years: 1.5	NA
	Correction osteotomy + Medial CMI (n=23)	NA	1 year: 79 2 years: 83	1 year: 90.4 2 years: 93.6	NA	1 year: 2.3 2 years: 2.2	NA
Lucidi et al. 2022 [36]	Medial/Lateral CMI (n=156)	NA	NA	NA	NA	NA	19 (12.2)
Monllau et al. 2011 [37]	Medial CMI (n=25)	NA	NA	1 year: 89.6 (5.5) 10 years: 87.5 (10.25)	NA	1 year: 1.5 (1.25) 10 years: 2 (1.5)	2 (8)
Reale et al. 2021 [39]	Medial/Lateral CMI (n=25)	NA	10 years: 62.1 (22.6)	NA	10 years: 3.8 (1)	10 years: 2.7 (2.4)	5 (20)
	Medial/Lateral Actifit (n=22)	NA	10 years: 67.4 (12.4)	NA	10 years: 3 (0.7)	10 years: 3.4 (2.5)	5 (22.7)
Rodkey et al. 2008 [40]	Acute medial CMI (n=75)	NA	NA	5 years: 90	NA	5 years: 0.5	5 (6.67)
	Acute control (n=82)	NA	NA	5 years: 87	NA	5 years: 0.6	5 (6.1)
	Chronic medial CMI (n=85)	NA	NA	5 years: 79	NA	5 years: 1.9	9 (11)
	Chronic control (n=69)	NA	NA	5 years: 78	NA	5 years: 2.1	18 (26)
Schenk et al. 2019 [41]	Medial/Lateral CMI (n=39)	NA	NA	1 year: 95 (6.5) 7 years: 91 (8)	1 year: 6 (4–9) 7 years: 6 (3–10)	1 year: 0.87 (1.4) 7 years: 2.1 (1.7)	NA
Spencer et al. 2012 [42]	Medial/Lateral CMI (n=9)	1 year: 49 2 years: 57	1 year: 65 2 years: 71.8	1 year: 80 2 years: 82.9	1 year: 4 2 years: 5.2	1 year: 82 2 years: 88.8	1 (11.1)
	Medial/Lateral Actifit (n=5)	1 year: 60 1.5 years: 61.4	1 year: 70 1.5 years: 74	1 year: 85 1.5 years: 86.6	1 year: 3.9 1.5 years: 4.4	1 year: 83 1.5 years: 85.6	0 (0)
Steadman and Rodkey 2005 [43]	Medial CMI (n=8)	NA	NA	1 year: 89.38 (9.5) 2 years: 91.5 (6.23) 5 years:88.12 (8.66)	1 year: 5 (3.75–5.25) 2 years: 5 (4–6.25) 5 years: 6 (5.5–7)	1 year: 0.71 (0.3) 2 years: 0.18 (0.18) 5 years: 1.09 (1.72)	NA
Whitehouse et al. 2016 [45]	MSC/Medial CMI (n=5)	NA	1 year: 66.2 (7.5) 2 years: 72.67 (15.16)	1 year: 83.3 (23.8) 2 years: 84 (22.74)		NA	2 (40)
Yoon et al. 2024 [46]	Medial/Lateral CMI (n=19)	1 year: 65 (19)	1 year: 80 (10)	NA	NA	1 year: 0.7 (1.1)	NA
	Medial/Lateral meniscectomy (n=14)	1 year: 64 (18)	1 year: 74 (14)	NA	NA	1 year: 0.9 (0.9)	NA
Zaffagnini et al. 2006 [10]	Medial CMI (n=8)	NA	NA	NA	NA	1 year: 1.3 (0.5) 2 years: 1.1 (0.4) 7 years: 1.8 (0.9)	NA
Zaffagnini et al. 2011 [47]	Medial CMI (n=17)	10 years: 54 (4)	NA	5 years: 92 (11) 10 years: 92 (12)	5 years: 4 (3.5–4.5) 10 years: 4 (2.5–5.5)	5 years: 1.5 (1) 10 years: 1.25 (0.8)	2 (11.76)
	Medial meniscectomy (n=16)	10 years: 43 (10)	NA	5 years: 81 (11) 10 years: 80 (9)	5 years: 4 (2.75–5.25) 10 years: 3 (1.75–4.25)	5 years: 2 (1.25) 10 years: 3.2 (1.8)	2 (12.5)
Zaffagnini et al. 2012 [49]	Lateral CMI (n=24)	2 years: 0.892 (0.14)	NA	2 years: 92.7 (13.8)	2 years: 5 (4-7)	2 years: 1.95 (2.56)	1 (4.17)
Zaffagnini et al. 2015 [48]	Lateral CMI (n=43)	NA	NA	1 year: 89.6 (9.8) 2 years: 93.2 (7.2)	1 year: 5 (4-6) 2 years: 5 (4-7)	1 year: 0.7 (1.3) 2 years: 0.3 (0.5)	5 (11.6)

Discussion

This systematic review was made with 27 studies involving 1,264 patients and evaluated three distinct collagen-based interventions for meniscal pathology: CMI, AMMR, and oral collagen supplementation. Each intervention demonstrated a distinct clinical profile with specific applications and outcomes. CMI demonstrated sustained functional improvement across extended follow-up periods, with consistent performance on validated outcome measures [10,40,48]. However, MRI evaluations revealed concerning structural findings, including predominantly partial implant resorption, frequent meniscal extrusion, and persistent hyperintense signal intensity [33,34,41,54]. AMMR demonstrated progressive functional improvement, with continuous advancement across all validated outcome measures, throughout extended follow-up periods [20,53]. MRI evaluations using WORMS showed predominantly favorable structural outcomes, with the majority of patients achieving excellent scores and demonstrating tissue integration [28]. However, some studies reported gradual score increases over time, particularly in combined AMMR+ACLR cases, although only a minimal number of cases progressed to an early osteoarthritis classification [20,53]. Oral collagen supplementation showed rapid and significant improvements in pain, quality of life, and functional capacity when directly compared to a placebo. Benefits were observed within the first month and sustained throughout the study period. On the other hand, neither of the included collagen supplementation studies incorporated MRI or structural imaging assessments. Limiting the ability to assess the differences in the structure compared to the surgical techniques [9,50]. Safety profiles varied by intervention complexity. CMI demonstrated variable failure rates ranging from 1.5% to 40% (averaging 11%-12%), while AMMR showed lower rates (4.17%-21.1%). Supplementation reported no adverse events or treatment failures during the study periods [9,10,18,20,32-54].

Our findings align with and extend previous systematic reviews examining meniscal scaffold interventions. A prior meta-analysis comparing CMI with Actifit in treating partial meniscal deficiencies found that both scaffolds demonstrated significant improvements in all clinical scores, with no significant differences between them in patient-reported outcome measures, activity levels, or failure rates [55]. However, Han and colleagues' systematic review presented a contrasting conclusion, finding no superiority in chondroprotective effects for either CMI Actifit compared to meniscectomy [56]. Regarding radiographic outcomes, our findings regarding CMI aligned with a previous umbrella review, which reported variable MRI results across included studies, with some demonstrating morphological deterioration of the implant. In contrast, others suggested possible chondroprotective effects - a pattern consistent with our observations [57].

Meniscal injuries represent a significant global health and economic burden. These injuries affect a range of 12%-14% of individuals worldwide, with an annual incidence of 61 cases per 100,000 people [58]. Approximately 850,000 annual cases make meniscal surgery one of the most frequent orthopedic procedures worldwide [59]. The annual costs of the procedures performed annually in the United States surpass $4 billion [60], representing direct medical expenditures for surgical intervention, hospitalization, and immediate postoperative care. However, the comprehensive economic impact is substantially greater when considering indirect costs, as the total injury economic burden reaches $4.2 trillion globally when including medical care, work loss, and quality of life impacts [61]. This substantial economic burden underscores the critical need for cost-effective treatment strategies.

In this economic context, the cost-effectiveness profiles of collagen-based interventions vary considerably. Linke et al. demonstrated that one of the main disadvantages is the cost of the implant and the implantation process [35]. CMI consistently demonstrates poor cost-effectiveness across international studies, with Dutch analyses reporting lifetime incremental cost-effectiveness ratios (ICERs) of €54,463 per quality-adjusted life-year (QALY), escalating to €297,727 per QALY over five years, while US studies showed costs of $73,445 per QALY (lifetime) and $401,492 per QALY (five years) [62]. Based on different sources, the standalone CMI would require 34% relative risk reduction to achieve cost-effectiveness thresholds - a benchmark current products fail to meet [62]. Conversely, AMMR demonstrates superior cost-effectiveness at €29,897-34,213 per QALY [63]. This collagen matrix wrapping technique proved cost-effective from both healthcare system and private patient perspectives [64]. Generating even more QALYs than meniscectomy despite higher upfront costs [64]. Despite the presentation of some cost-effectiveness data regarding CMI and AMMR, cost-effectiveness data for collagen supplementation remains absent, representing a critical knowledge gap, though it likely represents the least expensive option given its non-invasive nature [65,66].

Our study has several strengths, notably our inclusion of a relatively large population size for this topic, and we are the first to compare these three interventions. The quality of the included papers was relatively high, and we used three different methods to assess their quality carefully. The inclusion of extended follow-up studies enabled us to give reliable long-term insights. Our study also faced several limitations, including substantial heterogeneity in study design, patient populations, lesion characteristics, concomitant procedures, and follow-up durations, which precluded the conduct of a meta-analysis. Supplementation studies lacked MRI evaluation and had markedly shorter follow-up compared to surgical interventions. Absence of standardized protocols for intervention application, rehabilitation, and outcome timing introduces variability, complicating direct comparisons. Cost-effectiveness data were limited to CMI and AMMR from specific healthcare contexts, with no economic analyses for supplementation, which prevented us from estimating cost-effectiveness as a distinct outcome.

We recommend future research to conduct standardized multi-center RCTs with adequate sample sizes to compare interventions using uniform outcome measures and extended follow-up. Supplementation studies require MRI-based structural assessments, longer follow-up periods, larger sample sizes, and investigation of optimal dosing and formulations. Finally, we recommend developing predictive models that identify optimal candidates for each intervention based on tear and patient characteristics, thereby enhancing treatment selection.

## Conclusions

This systematic review suggests that collagen-based interventions offer viable options for meniscal pathology, with each suited to different clinical scenarios. CMI demonstrates sustained functional improvement over 10 years for structural replacement in partial deficiency, though with variable failure rates and concerning MRI findings of partial resorption and extrusion. AMMR demonstrates progressive functional enhancement with favorable structural outcomes for complex tears that require tissue preservation, resulting in lower failure rates and superior cost-effectiveness. Oral supplementation provides rapid symptomatic relief within one to three months for conservative symptom management, with no reported adverse events, though long-term structural effects remain unestablished. However, these findings must be interpreted cautiously due to the absence of direct comparative studies and substantial heterogeneity across included trials. Future research should prioritize head-to-head randomized controlled trials with standardized protocols, extended follow-up for supplementation studies, including MRI assessments, and the development of predictive models to guide personalized treatment selection based on patient and lesion characteristics.

## Appendices

Full detailed search strategy 

**Table 6:** Full detailed search strategy

Database	Search Query	Number of Records Retrieved
PubMed	("collagen"[Supplementary Concept] OR "collagen"[All Fields] OR "collagen"[MeSH Terms] OR "collagens"[All Fields] OR "collagen s"[All Fields] OR "collagenation"[All Fields] OR "collagene"[All Fields] OR "collageneous"[All Fields] OR "collagenic"[All Fields] OR "collagenization"[All Fields] OR "collagenized"[All Fields] OR "collagenous"[All Fields] OR ("collagen"[Supplementary Concept] OR "collagen"[All Fields] OR "avicon"[All Fields] OR "collagen"[MeSH Terms]) OR ("collagen"[Supplementary Concept] OR "collagen"[All Fields] OR "avitene"[All Fields] OR "collagen"[MeSH Terms]) OR ("collagen"[Supplementary Concept] OR "collagen"[All Fields] OR "collastat"[All Fields] OR "collagen"[MeSH Terms]) OR ("collagen"[Supplementary Concept] OR "collagen"[All Fields] OR "dermodress"[All Fields] OR "collagen"[MeSH Terms]) OR ("collagen"[Supplementary Concept] OR "collagen"[All Fields] OR "pangen"[All Fields] OR "collagen"[MeSH Terms]) OR ("collagen"[Supplementary Concept] OR "collagen"[All Fields] OR "zyderm"[All Fields] OR "collagen"[MeSH Terms]) OR "Instat"[All Fields] OR "Helistat"[All Fields] OR "Collatamp"[All Fields] OR "Fibracol"[All Fields] OR "Puracol"[All Fields] OR "Promogran"[All Fields] OR "Biopad"[All Fields] OR ("collacote"[Supplementary Concept] OR "collacote"[All Fields]) OR "CollaPlug"[All Fields] OR "CollaTape"[All Fields] OR ("glutaraldehyde cross linked collagen"[Supplementary Concept] OR "glutaraldehyde cross linked collagen"[All Fields] OR "zyplast"[All Fields]) OR ("artecoll"[Supplementary Concept] OR "artecoll"[All Fields]) OR ("cymetra"[Supplementary Concept] OR "cymetra"[All Fields]) OR ("bio gide"[Supplementary Concept] OR "bio gide"[All Fields]) OR "Periocol"[All Fields] OR "OsseoGuard"[All Fields] OR "Verisol"[All Fields] OR "Fortigel"[All Fields] OR "Peptan"[All Fields]) AND ("meniscopathies"[All Fields] OR "meniscopathy"[All Fields] OR "Meniscal disease"[All Fields] OR "Meniscal pathology"[All Fields] OR "Meniscal lesion"[All Fields] OR "Meniscal disorder"[All Fields] OR "Meniscal degeneration"[All Fields] OR "degenerative menisc*"[All Fields] OR "meniscal injur*"[All Fields] OR "meniscus tear*"[All Fields] OR "meniscal tear*"[All Fields] OR "meniscal defect*"[All Fields] OR "Torn meniscus"[All Fields] OR "Meniscal derangement"[All Fields] OR ("meniscal"[All Fields] AND ("syndrome"[All Fields] OR "syndromes"[All Fields])) OR "meniscus implant*"[All Fields] OR "meniscal implant*"[All Fields])	360
Scopus	TITLE-ABS-KEY((Collagen OR Avicon OR Avitene OR Collastat OR Dermodress OR Pangen OR Zyderm OR Instat OR Helistat OR Collatamp OR Fibracol OR Puracol OR Promogran OR Biopad OR CollaCote OR CollaPlug OR CollaTape OR Zyplast OR Artecoll OR Cymetra OR Bio-Gide OR Periocol OR CollaGuide OR OsseoGuard OR Verisol OR Fortigel OR Peptan) AND (Meniscopathy OR "Meniscal disease" OR "Meniscal pathology" OR "Meniscal lesion" OR "Meniscal disorder" OR "Meniscal degeneration" OR "Degenerative menisc*" OR "Meniscal injur*" OR "Meniscus tear*" OR "Meniscal tear*" OR "Meniscal Defect*" OR "Torn meniscus" OR "Meniscal derangement" OR "Meniscal syndrome" OR "Meniscus implant*" OR "Meniscal implant*"))	504
Web of Science	ALL=((Collagen OR Avicon OR Avitene OR Collastat OR Dermodress OR Pangen OR Zyderm OR Instat OR Helistat OR Collatamp OR Fibracol OR Puracol OR Promogran OR Biopad OR CollaCote OR CollaPlug OR CollaTape OR Zyplast OR Artecoll OR Cymetra OR Bio-Gide OR Periocol OR CollaGuide OR OsseoGuard OR Verisol OR Fortigel OR Peptan) AND (Meniscopathy OR “Meniscal disease” OR “Meniscal pathology” OR “Meniscal lesion” OR “Meniscal disorder” OR “Meniscal degeneration” OR “Degenerative menisc*” OR “Meniscal injur*” OR “Meniscus tear*” OR “Meniscal tear*” OR “Meniscal Defect*” OR “Torn meniscus” OR “Meniscal derangement” OR “Meniscal syndrome” OR “Meniscus implant*” OR “Meniscal implant*”))	426
Cochrane Library	((Collagen OR Avicon OR Avitene OR Collastat OR Dermodress OR Pangen OR Zyderm OR Instat OR Helistat OR Collatamp OR Fibracol OR Puracol OR Promogran OR Biopad OR CollaCote OR CollaPlug OR CollaTape OR Zyplast OR Artecoll OR Cymetra OR Bio-Gide OR Periocol OR CollaGuide OR OsseoGuard OR Verisol OR Fortigel OR Peptan) AND (Meniscopathy OR “Meniscal disease” OR “Meniscal pathology” OR “Meniscal lesion” OR “Meniscal disorder” OR “Meniscal degeneration” OR “Degenerative menisc*” OR “Meniscal injur*” OR “Meniscus tear*” OR “Meniscal tear*” OR “Meniscal Defect*” OR “Torn meniscus” OR “Meniscal derangement” OR “Meniscal syndrome” OR “Meniscus implant*” OR “Meniscal implant*”)):ti,ab,kw	21
Embase	(collagen:ab,ti OR avicon:ab,ti OR avitene:ab,ti OR collastat:ab,ti OR dermodress:ab,ti OR pangen:ab,ti OR zyderm:ab,ti OR instat:ab,ti OR helistat:ab,ti OR collatamp:ab,ti OR fibracol:ab,ti OR puracol:ab,ti OR promogran:ab,ti OR biopad:ab,ti OR collacote:ab,ti OR collaplug:ab,ti OR collatape:ab,ti OR zyplast:ab,ti OR artecoll:ab,ti OR cymetra:ab,ti OR 'bio gide':ab,ti OR periocol:ab,ti OR collaguide:ab,ti OR osseoguard:ab,ti OR verisol:ab,ti OR fortigel:ab,ti OR peptan:ab,ti) AND (meniscopathy:ab,ti OR 'meniscal disease':ab,ti OR 'meniscal pathology':ab,ti OR 'meniscal lesion':ab,ti OR 'meniscal disorder':ab,ti OR 'meniscal degeneration':ab,ti OR 'degenerative menisc*':ab,ti OR 'meniscal injur*':ab,ti OR 'meniscus tear*':ab,ti OR 'meniscal tear*':ab,ti OR 'meniscal defect*':ab,ti OR 'torn meniscus':ab,ti OR 'meniscal derangement':ab,ti OR 'meniscal syndrome':ab,ti OR 'meniscus implant*':ab,ti OR 'meniscal implant*':ab,ti)	499
